# Manufacturing protocol and post processing of ultra-thin gas diffusion layer using advanced scanning techniques

**DOI:** 10.1038/s41598-024-63751-z

**Published:** 2024-06-07

**Authors:** Hossein Pourrahmani, Jan Van Herle

**Affiliations:** 1 Group of Energy Materials, École Polytechnique Fédérale de Lausanne, 1951 Sion, Switzerland; 2 University of California, Irvine, California 92617 USA

**Keywords:** Proton exchange membrane fuel cells (PEMFC), Gas diffusion layer (GDL), Low thickness, Water/thermal management, Fuel cells, Materials for energy and catalysis, Computational methods

## Abstract

The typical commercial size of a Gas Diffusion Layer (GDL) for Proton Exchange Membrane Fuel Cell (PEMFC) application is around 180 μm up to 290 μm. GDL facilitates the diffusion of reactants to the catalyst layers and liquid removal from the membrane to the flow field. In this regard, GDL should be a porous region with conductive materials as thin as possible to reduce the size and the costs. Lowering the thickness of the GDL also results in better performance of the stack since it increases the speed of reactants to reach the catalysts. However, the main obstacle is the formation of ultra-thin porous GDL, which can be also named as standalone microporous layer (MPL). The novelty of this study is the manufacturing process and production of ultra-thin porous GDL with carbon and Polytetrafluoroethylene (PTFE) as the main materials. The produced GDL has the thickness of 28.9 μm, which has been measured using microscope imaging. This novel GDL can be used as the conductive diffusive region inside the PEM fuel cells, Alkaline fuel cells, and the cathode of PEM and Alkaline electrolyzers. Additionally, the novel invention can be considered as a 2D membrane for carbon capture purposes after being functionalized.

## Introduction

Considering the usages of Proton Exchange Membrane Fuel Cell (PEMFC) in mobility applications^1,2^, performance improvement of this electrochemical device is of importance to the authorities. The most reliable solution for the diffusion of reactants to the membrane and the removal of water to the flow fields in PEMFCs is to use GDL and Microporous Layer (MPL) in a way that the MPL is located between the GDL and the catalyst layer (CL)^3^. The main responsibilities of the GDL are to facilitate the flow of the reactants to the CL, the flow of water from the membrane to the flow fields, and the electrical/thermal conductivities^4^. Due to the porous nature of the ultra-thin GDL, which can be also considered as the standalone MPL, all the above-mentioned responsibilities of the GDL in a PEMFC can be provided^5^. In this sub-section the relevant state of the art will be discussed.

**Figure 1:**
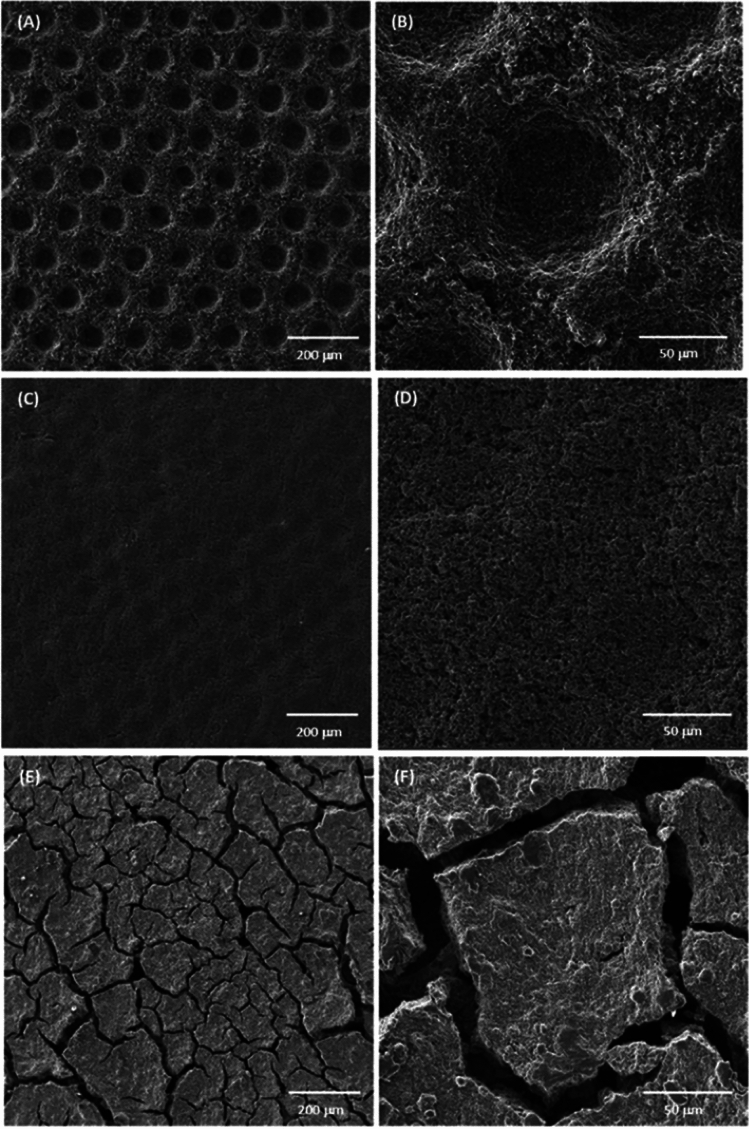
Samples by Hendricks et al.^6^. (**A**,**B**) sa-MPLs imprinted on the metallic GDLs; (**C**,**D**) Wet coated MPLs imprinted on the metallic GDLs; (**E**,**F**) sa-MPLs not being coated on any substrate. (Copyright license number 5545911256208 issued on 11th May 2023).

The most similar study to manufacture the ultra-thin GDL, that is called standalone MPL (sa-MPL) in that study, was developed by Hendricks et al.^6^. The demonstrated results in Hendricks et al.^6^ showed that the sa-MPL is difficult to be produced due to the low thickness and it will result in the formation of the cracks in the microstructure (see Fig. 1). The usage of metallic GDLs (MGDLs) inside the PEMFC results in better thermal/electrical conductivities in addition to better mechanical support for the MEA. However, MGDLs have limitations in gas transport due to the anisotropic structure that result in the non-homogeneous gas/catalyst electrochemical reaction in addition to leading to higher thickness and weight of the cell. The integration of the sa-MPL to the MGDL results in the better mass transport properties of the MGDL, however, higher thickness and weight of the cell remained unsolved. At the current stage, the PEMFC should be as light and as small as possible while the performance is in the highest condition to accelerate the commercialization.

The developed study by Sarker et al.^7^, showed the importance of the percentages of the utilized materials inside the MPL to increase liquid water removal from the stack. The main component of the GDL and MPL is carbon fiber to act as the mechanical support for the Membrane Electrode Assembly (MEA) and to increase the thermal/electrical conductivity. Although the electrical conductivity of the GDL will be improved using the carbon fibers, GDL is also supposed to act as a medium to remove the liquid water, hence it should be benefited from hydrophobic properties. In this regard, different types of binders had been used and the scientific community is almost certain that the Polytetrafluoroethylene (PTFE) is the best option to act as a binder between the carbon fibers to increase the hydrophobicity of the GDL/MPL with the cost of lower conductivities. Sarker et al.^7^ analyzed that although adding higher PTFE may result in better hydrophobic conditions, the increased PTFE loading is not enough to prevent the flooding inside the cell. In this regard, the usage of PTFE should be selected deliberately to prevent flooding/drying out the membrane and/or reduce conductivity.

The liquid water removal from the GDL and MPL is a function of the capillary pressure, which is a function of the wettability and the microstructure of the utilized domain. In this regard, Forner-Cuenca et al.^8,9^ suggested manufacturing of a hydrophobic and hydrophilic regions to create controlled water columns inside the GDL. However, this idea has serious difficulties toward manufacturing and results in more corrosion in the hydrophobic conditions that reduce the lifetime of the cell. Additionally, this concept of the GDL lead to non-homogenous distribution of reactants in the GDL, which results in non-uniform distribution of the reactants in the CL for the electrochemical reactions. In this regard, although the idea of hydrophobic/hydrophilic GDL may increase the voltage in short term, the disadvantages have prevented it to be commercially viable.

The results of the study by Philips et al.^10^ enabled to identify the main important parameters on the manufacturing of the GDL to increase diffusion rate. Among 26 synthesized electrodes, the main impactful manufacturing factors were identified and prioritized. The results were in-line with the study by Sarker et al.^7^ regarding the PTFE percentage in addition to the rolling thickness of the GDL. The temperature and the pressure were also recognized as the integral factors to tune the GDL characteristics. Other influential parameters such as compression of the GDL was studied by Lin et al.^11^. The results indicated that GDL’s compression led to the decrease in the gas permeability and contact resistance.

The utilization of the carbon nanotubes (CNTs) was also evaluated as an efficient method to increase the performance of the GDLs^12^, however, the high cost of the CNTs have been always a salient factor to prevent them for commercialization purposes. As an alternative to the common methodologies to manufacture the GDL, Taherian et al.^13^ omitted the carbonization and graphitization steps of the carbon fibers using expanded graphite as filler to increase the conductivity. The results indicated similar performance to Toray GDLs while benefiting from lower costs.

### Novelties of the current study

This section introduces an inventive process of manufacturing GDLs that can be used instead of conventional methods to reduce the thickness of GDL layers, hence the PEMFC stack, to improve the water/thermal management, and to reduce costs. The novel manufacturing method enables the production of ultra-thin GDL without any defects and cracks with uniform porosity throughout the sample. The measurements on conductivity, permeability, and other microstructural properties verified the high performance of this novel ultra-thin GDL, which can be used as the conductive diffusive region inside PEM fuel cells, alkaline fuel cells, and the cathode of PEM and alkaline electrolyzers. Additionally, this component can be used as standalone MPL in the case of using metallic GDLs.

## Methodology and experimental procedure

This section is the main novelty of this study, which enables the production of ultra-thin GDLs. Here, ultra-thin GDL refer to GDLs with the thickness of less than 100 μm. In this study, the thickness of the 28.9 μm was reached; however, it is possible to reach other thicknesses than 28.9 μm with the same methodology. The novel methodology enables the production of GDL with lower thickness of 100 μm:

- The utilized material for this study is the commercially available MPL inks and binders (this study used PTFE layers). There are no limitations on the thickness of the PTFE layers or, the PTFE shape (it can be in the form of layer or other forms). The PTFE in this study has been used to improve the formation of the ultra-thin GDL.
- The MPL inks are available commercially. MPL inks are usually a combination of the carbon fibers and the binders. MPL inks may be called as coating material for GDL or any other names as well. In this regard, all types of solutions with carbon fibers and other extra materials (such as binders, etc.) can be considered as the MPL ink.
- To make the ultra-thin GDL, an oven, a glass (resisting temperatures higher than 250 Celsius degrees), a coating device, and a detacher to perform the skiving are required. In this study, a sharp blade was used to perform the skiving. For the coating, this work used a Doctor blade coating machine. The oven should be able to reach temperatures higher than 250 Celsius degrees.
- To make the ultra-thin GDL, MPL ink should be coated on the glass using the coating machine. The thickness of the ultra-thin GDL can be controlled in this condition with the amount of the utilized MPL ink and the thickness that has been given to the coating machine.
- Once the coating is done, the PTFE layer (material) should be located on the same glass. It should be noted that the MPL ink, which has been coated on the glass, has already PTFE or other types of binder and the PTFE layer (material) is not supposed to located on the coating. The PTFE layer (material) should be located on the same glass but not on the coating. The PTFE layer is also separate from the coating and it’s completely dependent from it. The reason for putting the PTFE layer is the evaporation and condensation in the oven to fill out the possible appearing cracks. There have been many studies to produce standalone MPL or ultra-thin GDL as shown in Fig. 1, however, the layer does not form or it forms with cracks. The positioning of the PTFE layer and the subsequent evaporation and condensation in the oven prevent the formation of cracks and fills out the possible upcoming cracks with condensed PTFE.
- The coated MPL ink should spend some pre-heating time. The pre-heating temperature can be any temperature less than 100 Celsius degrees. In this study, the room temperature was used for pre-heating temperature. Additionally, the time can be anything more than zero seconds. This study used the pre-heating time of 30 min.
- The formation of the ultra-thin GDL happens in the oven. The sintering temperature of the used MPL ink was 350 Celsius degrees, and the temperature of the oven was selected 380 Celsius degrees. However, the temperature is highly dependent on the type of MPL ink that has been used and the temperature of the oven should be higher than the sintering temperature of the MPL ink, in this case 380 Celsius degrees. Lower temperature of the oven than the sintering temperature prevents the formation of the ultra-thin GDL.
- It should be noted that the oven should have been already turned on and reach to the selected oven temperature. As mentioned, oven temperature is a selection of the operator based on the sintering temperature. The important point is that the MPL ink should experience the oven temperature in one sudden change, which is reaching from pre-heating temperature to the oven temperature.
- Regarding the existing binder on the glass, as the evaporation temperature of the binder is usually lower than the MPL ink, reaching the sintering temperature makes the binder (PTFE layer) to be evaporated in the oven. Once the evaporation happens, the existing particles of the binder will spread in the oven and some will be located on the MPL ink. In this regard, the condensation of the MPL ink will have more binder as it was before, however, this amount wouldn’t be too much to have adverse impacts on the electrical conductivity. The high amount of binder in the MPL ink results in low electrical conductivities, in this regard, the manufacturing companies of the MPL inks usually don’t add considerable amount of them. However, with this methodology, the binder will not be too much and will be sufficient enough to improve the formation for low thicknesses while having high electrical conductivities. The reason for this claim is the fact that insufficient amount of PTFE will prevent the formation of ultra-thin GDL, hence instead of having a layer, there will be powders or agglomerated particles of MPL ink. In the next sections of this study, the electrical conductivity of the novel ultra-thin GDL was measured and the suitability of the invention was proved.
- It should be noted that the current methodology also includes any type of methodology to add small amount of binder on the MPL ink without reducing the electrical conductivity.
- The MPL ink should remain in the oven for a definite sintering time. Sintering time may be different based on the used MPL ink and the operator preference. This study used the sintering time of 50 min.
- After sintering, the sample should be cooled. The cooling temperature is an operator preference just like the stages of the cooling. This study used the room temperature for the cooling temperature and the process was done in one stage.
- Detaching of the ultra-thin GDL can be done after cooling using skiving method. The provided area of the ultra-thin GDL is also dependable on the operator/researcher. With this methodology it is possible to reach real size areas that is being used for commercial applications (like 40 cm^2^ $\times$ 40 cm^2^).

The surface quality of the ultra-thin GDL was first analyzed using the Scanning Electron Microscopy (SEM) using the obtained signals from the Everhart–Thornley secondary electron secondary ion (SESI) detector. The Electron high tension was selected 22 kV to facilitate the detection of existing elements inside the sample using the Energy Dispersive X-ray spectrometry (EDXS). The EDXS analysis also determines the percentage of the used composition inside the sample which enables the theoretical calculation of the conductivity.

The three-dimensional geometry of the sample was also analyzed using the micro computed tomography ($\mu$-CT) scanning images, which is a non-destructive method and determines the details of the geometry. The performed $\mu$-CT scanning for the ultra-thin GDL had the image resolution of 1.08684 μm at the acceleration voltage of 40 kV with 12 averaging frames at corressponding current and exposure time of 120 μA and 500 ms. The rotation step and the stage temperature were 0.22 degrees and 21 °C, respectively. Using the $\mu$-CT images, the microstructural properties including the permeability, porosity, conductivity, etc. were obtained using the commercial software^14^. The $\mu$-CT images also enable the simulation of the flow field^15^ inside the ultra-thin GDL; thus, the distribution of the water columns inside the domain.

Regarding the calculation of the microstructural properties, the utilized method in this study is based on using EDXS to understand the composition of the material and SEM and $\mu$-CT images to understand the shape and the size of the sample. The microstructural properties can be later calculated using the Xlab module of Avizo software. Due to the high precision of the EDXS to understand the composition and the one micrometer resolution of the $\mu$-CT, the accuracy and the precision of the current method is much higher than the available experimental methods such as 4-point probe to calculate the conductivity. In this regard, the authors highly recommend the current method as a reference for future calculation of the microstructural properties.

## Governing equations and the calculation of the microstructural properties

### Absolute permeability

The ability of a porous material to transmit a single-phase fluid is defined as absolute permeability, which is a material’s property. Although the common unit is (${m}^{2}$), permeability can be shown by darcy (d), which is equal to 1d = 0.986923 μm^2^. Absolute permeability can be calculated using Darcy’s law as follows ^16^:

1 $$\frac{Q}{S}=-\frac{k}{\mu }\frac{\Delta P}{L}$$

where, $Q ({\text{m}}^{3}/\text{s})$ is the mass flow rate, $S ({\text{m}}^{2})$ is the corresponding cross-section that the single-phase fluid passes through, $k ({\text{m}}^{2})$ is the absolute permeability, $\mu$ (Pa s) is the dynamic viscosity, $\Delta P$ (Pa) is the gradient pressure difference, and $L \left(\text{m}\right)$ is the length of the sample. It should be noted that in multi-phase flows the relative permeability should be calculated while single-phase fluids correspond to the absolute permeability and can be calculated using the Stokes equations^17^:

2 $$\left\{\begin{array}{c}\overline{\nabla }.\overline{V }=0\\ \mu {\nabla }^{2}\overline{V }-\overline{\nabla }P=0\end{array}\right.$$

here, $\overline{\nabla }.$ is the divergence operator, while $\overline{\nabla }$ is the gradient operator. $\overline{V }$ is the velocity, P is the pressure, and ${\nabla }^{2}$ is the Laplacian operator. Assuming incompressible + Newtonian fluid, the density and dynamic viscosity will be assumed to be constant. Additionally, the flow is steady-state and laminar, which results in not changing the velocity over time and not having turbulence. Using the volume averaged method and transforming Eq. (2) to a tensorial problem, the permeability tensor can be calculated as follows^18^:

3 $$\stackrel{-}{\overline{k} }=\frac{1}{v}{\int }_{v}\stackrel{-}{\overline{D} }dv$$

where, $\stackrel{-}{\overline{D} }$ is the velocity perturbation field and $v$ is the analyzing volume. The calculation of the permeability tensor enables the determination of the permeability along any direction in space in addition to the anisotropy of a porous medium. Using the obtained permeability, the tortuosity ($\tau$) can be calculated using the porosity ($\epsilon$) as follows:

4 $$k=\frac{{\epsilon }^{3}}{{\left(1-\epsilon \right)}^{2}}\frac{{\tau }^{2}}{8}$$

It should be noted that corresponding boundary conditions should be considered to calculate the absolute permeability^19^:

- No-slip flow condition at the fluid–solid interface to use the Stokes equations (Eq. 2). If the Lattice Boltzmann Method (LBM) is used, this boundary condition is not needed to be considered.
- The selection of input/output pressures, and the flow rate.
- If the obtained image of the solid phase is not perpendicular to the main flow of the fluid, one-voxel-wide plane is added considering the no-slip boundary condition. In other words, facilitating the detection of the sample from outside by isolating the solid phase from external flows outside the domain.

### Molecular diffusion

Based on Fick’s first law, molecular diffusion is the passive transportation of a dissolved mass from a higher chemical energy state to a lower chemical energy state by random molecular motion. In this regard, Fick’s law can describe the diffusion of species in a free solution as follows:

5 $$\overline{j }=-D.\overline{\nabla }c$$

where, $\overline{j } (\frac{mol}{{m}^{2}.s})$ is the mass flux, while $D \left(\frac{{m}^{2}}{s}\right)$ and $c \left(\frac{mol}{{m}^{3}}\right)$ are the diffusion coefficient and the concentration, respectively.

The transient diffusion in a porous medium can be described using Fick’s second law as follows:

6 $$\frac{\partial c}{\partial t}-D.{\nabla }^{2}c=0$$

Considering two reservoirs with a volume of ${V}_{R}$ and different initial concentrations of ${C}_{in}(t)$ and ${C}_{out}(t)$, Fick’s second law determines the diffusion using the following equations:

$${V}_{R}\frac{\partial {C}_{in}\left(t\right)}{\partial t}=D{\int }_{{S}_{in}}\overline{\nabla }c.\overline{n }dS$$

7 $${V}_{R}\frac{\partial {C}_{out}\left(t\right)}{\partial t}=D{\int }_{{S}_{out}}\overline{\nabla }c.\overline{n }dS$$

where, ${S}_{in}$ and ${S}_{out}$ are the cross-sections of the sample in which the reservoirs are connected. After experiencing the initial transient phase of diffusion, an established state will be obtained that $\frac{\partial {C}_{in}\left(t\right)}{\partial t}=-\frac{\partial {C}_{out}\left(t\right)}{\partial t}$. In this condition, the concentrations will evolve until they reach the equilibrium state of ${c}_{\infty }$. Assuming constant coefficients of $p$ and ${\lambda }^{2}$, the difference in the concentrations can be obtained:

8 $${C}_{out}\left(t\right)-{C}_{in}\left(t\right)=p.\text{exp}(-{\lambda }^{2}t)$$

Concentration can be also calculated at the position of $X$ and time of $t$ with a constant parameter of $A$ as follows:

9 $$c\left(X,t\right)=A\left[\left[\frac{\text{cos}\left(\frac{\lambda }{\sqrt{{D}_{app}}}-1\right)}{\text{sin}\left(\frac{\lambda }{\sqrt{{D}_{app}}}\right)}\right]\text{cos}\left(\frac{\lambda }{\sqrt{{D}_{app}}}X\right)+\text{sin}\left(\frac{\lambda }{\sqrt{{D}_{app}}}X\right)\right]\text{exp}\left(-{\lambda }^{2}t\right)+{c}_{\infty }$$

Taking into account $\frac{\partial {C}_{in}\left(t\right)}{\partial t}=-\frac{\partial {C}_{out}\left(t\right)}{\partial t}$, Eq. (9) changes to:

10 $$-\frac{{\lambda }^{2}\text{cos}\left(\frac{\lambda }{\sqrt{{D}_{app}}}-1\right)}{\text{sin}\left(\frac{\lambda }{\sqrt{{D}_{app}}}\right)}={D}_{app}\beta \frac{\lambda }{\sqrt{{D}_{app}}}$$

where, $\beta =\frac{{V}_{void space}}{{V}_{R}}$ is the ratio of the volume of the pores to the reservoir, and ${D}_{app}$ is the apparent diffusivity. Using a closure variable $\overline{b }$ to state the concentration pertubation (${\nabla }^{2}\overline{b }=0$), the dimensionless diffusivity tensor ($\stackrel{-}{\overline{D} }$) can be calculated as follows:

11 $$\epsilon \frac{\stackrel{-}{\overline{D}}}{{D }_{solution}}=\epsilon (\stackrel{-}{\overline{I} }+\frac{1}{{V}_{f}}{\int }_{{S}_{fs}}\overline{{n }_{fs}}\overline{b }ds)$$

here, $\epsilon$ is the porosity, ${D}_{solution}$ is the bulk solution diffusivity, ${V}_{f}$ is the volume of fluid, ${S}_{fs}$ is the area of the fluid–solid interface, and $\overline{{n }_{fs}}$ is the vector normal to the fluid–solid interface with the boundary condition of $-\overline{{n }_{fs}}.{\overline{\nabla } }_{c}=0$.

The boundary conditions of the inlet and the outlet are also a parameter of the concentrations as follow:

$${V}_{r}\frac{\partial {C}_{in}}{\partial t}={\int }_{{S}_{in}}\overline{{n }_{{S}_{in}}}.\overline{\nabla }c ds$$

12 $${V}_{r}\frac{\partial {C}_{out}}{\partial t}=-{\int }_{{S}_{out}}\overline{{n }_{{S}_{out}}}.\overline{\nabla }c ds$$

### Electrical conductivity

In this study, the electrical conductivity, which is the transportation of the electrical charges by an electrical field, has been calculated using Ohm’s law as follows:

13 $$\overline{i }=-\sigma \overline{\nabla }\nu$$

where, $\overline{i }$ ($A/{m}^{2}$) is the current density, $\sigma$ ($S/m$) is the electrical conductivity, and $\nu$ (V) is the electrical potential.

In this study to calculate the electrical conductivity, the porous region is considered to be only one solid phase (as a composition of different elements that the percentages are determined using EDXS). Considering the conservation of charge:

14 $$\overline{\nabla }.\overline{i }=0$$

As it is assumed to have a homogeneous phase in the ultra-thin GDL, $\sigma$ will not change in the space, which leads to:

15 $${\nabla }^{2}\nu =0$$

Using Ohm’s law on the entire porous region, the apparent electrical conductivity can be calculated using the following equation:

16 $$\frac{{i}_{total}}{S}=\sigma \frac{{V}_{in}-{V}_{out}}{L}$$

where, ${i}_{total}$ is the total electrical flux, $S$ is the cross-section area, $L$ is the length of the material, and ${V}_{in}/{V}_{out}$ are the input/output potentials. With the local implementation of Ohm’s law, ${i}_{total}$ can be calculated as follows:

17 $${i}_{total}={\int }_{S}-{\sigma }_{solution}\overline{\nabla }\nu .d\overline{s }$$

It should be noted that the inverse tensor of the conductivity tensor is the Formation Factor. The scalar format of the formation factor (F) is the average value of the eigenvalues and can validate the results using the following relation by Berryman et al.^20^

18 $$F=\frac{2}{\epsilon \left(1+\epsilon \right)}$$

### Thermal conductivity

Thermal conductivity is the ability of a material to transfer heat from a heat source to a sink source. Fourier’s law (Eq. 19) presents heat conduction for a homogeneous material^21^:

19 $$\overline{\varphi }=-\lambda \overline{\nabla }T$$

where, $\overline{\varphi }$($\frac{W}{{m}^{2}}$) is the heat flux, while $\lambda (\frac{W}{mK})$ and $T\left(K\right)$ are the thermal conductivity and the temperature of the material, respectively. In a non-homogeneous material with multiple phases, the transient heat conduction for the phase $\alpha$ is as follows^22^:

20 $${\left(\rho {c}_{p}\right)}_{\alpha }\frac{\partial {T}_{\alpha }}{\partial t}-{\lambda }_{\alpha }{\nabla }^{2}{T}_{\alpha }=0$$

here, ${\left(\rho {c}_{p}\right)}_{\alpha } \left(\frac{J}{{m}^{3}K}\right)$, ${\rho }_{\alpha } \left(\frac{kg}{{m}^{3}}\right)$, and ${c}_{{P}_{\alpha }} (\frac{J}{kg.K})$ are the heat capacity, density, and specific heat of the $\alpha$ phase, respectively. In the steady-state condition, the following equation should be considered:

21 $${\lambda }_{\alpha }{\nabla }^{2}{T}_{\alpha }=0$$

By applying a constant stream of heat between two opposite faces (${T}_{in}$ and ${T}_{out}$), and assuming the other faces as the thermal insulators, Fourier’s law can be implemented on the entire volume as follows :

22 $$\frac{{\varphi }_{total}}{{S}_{in}}=\lambda \frac{{T}_{in}-{T}_{out}}{L}$$

where, ${\varphi }_{total}$ is the total heat flux through the input face, while ${S}_{in}$ is the area of the input face, and $\lambda$ is the apparent thermal conductivity. By controlling the temperatures of ${T}_{in}$ and ${T}_{out}$, the thermal conductivity and the total heat flux can be calculated:

23 $${\varphi }_{total}={\int }_{{S}_{in}}-{\lambda }_{\alpha }\overline{\nabla }{T }_{\alpha }.d\overline{s }$$

## Results and discussion

**Figure 2:**
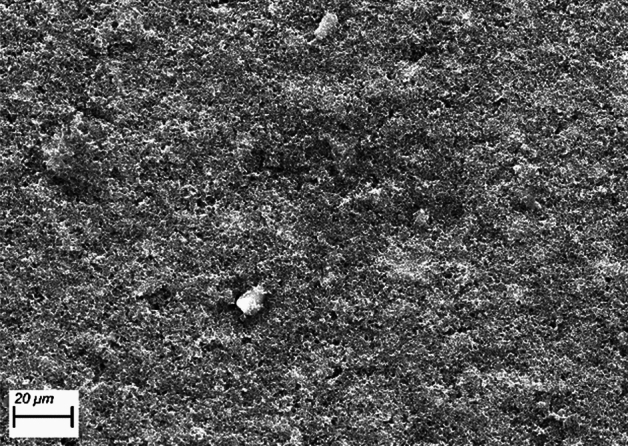
Obtained SEM image of the ultra-thin GDL. The image has been obtained for a working distance of 6.6 mm and acceleration voltage of 22 kV to allow for EDXS analysis while the probe’s current density was 3 nA.

In this section, the suitability and advantages of the current suggested ultra-thin GDL is explained. Figure 2 shows the obtained SEM image of the prepared ultra-thin GDL. As it can be seen, the surface morphology of the ultra-thin GDL is homogenous and has small pores that enable the transport of reactants and the removal of liquid water. The advantageous part about the ultra-thin GDL is that the size of the pores is quite small (maximum 2 μm based on Fig. 2). Small pore size enables lower velocities of the reactants and decreases the possibility of creating water columns inside the GDL. The formation of water columns inside the GDL is considered a deteriorating phenomenon since it degrades the pores more quickly and increases the possibility of ice formation in sub-zero temperatures that lead to difficulties for cold-start. Regarding the velocity in the ultra-thin GDL, lower velocities due to smaller pore sizes are considered advantageous since the thickness of the manufactured sample is low (28.9 μm in comparison to 170 ~ 200 μm of the commercial GDLs). In this regard, although the thickness of the ultra-thin GDL is low, the reactants will have enough time for diffusion and improvement of the electrochemical reactions.

In other words, it is believed that small pore sizes and homogeneously distributed pores throughout the GDL is a beneficial microstructure. However, the main problem so far was the manufacturing process. The reason is that for very low thicknesses, small pore sizes, and distributed pores results in the formation of cracks and even not forming the microstructure as a layer (but forming as powders). The main novelty of this section is the manufacturing process that enabled the formation of this layer without cracks with acceptable microstructural properties. It should also be noted that the ultra-thin GDL is a composition of carbon fiber and PTFE to have high conductivity and hydrophobicity at the same time.

Once the SEM image is obtained, it is possible to calculate the composition of the ultra-thin GDL to calculate the $\sigma$ ($\text{S}/\text{m}$), i.e. electrical conductivity. Based on the given composition for the ultra-thin GDL in Table 1, the electrical conductivity of the composition is $\sigma =1.5506\times {10}^{6}\text{ S}/\text{m}$.

**Table 1:** Corresponding composition of the ultra-thin GDL.

Element	Ultra-thin GDL (wt%)
Carbon (C)	63.12
Oxygen (O)	10.08
Fluorine (F)	25.43
Potassium (K)	0.07
Nickel (Ni)	0.79
Copper (Cu)	0.5

**Figure 3:**
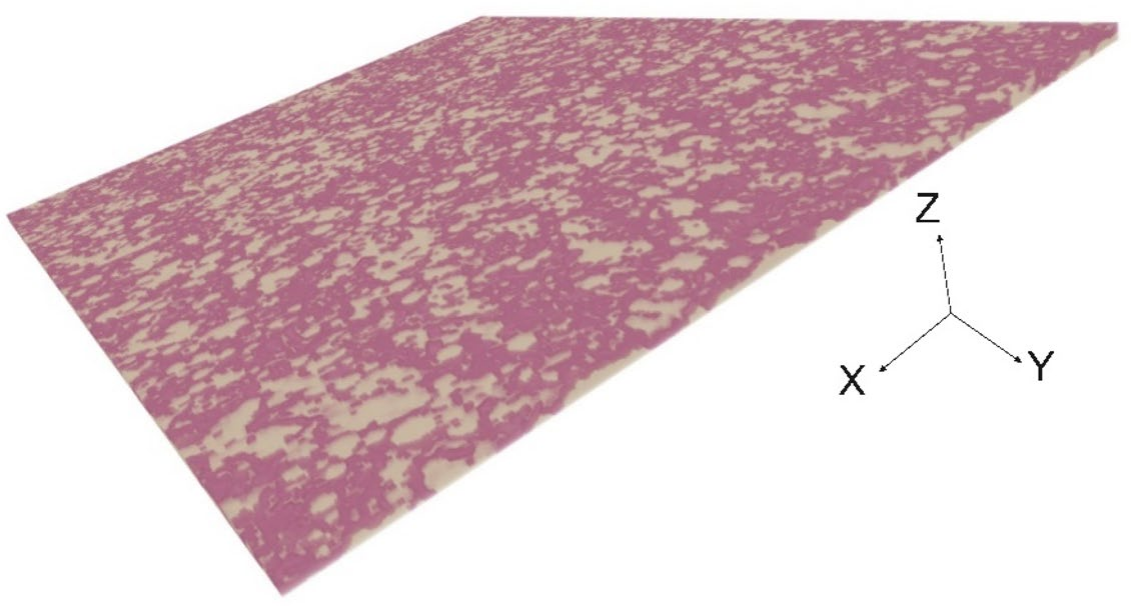
Segmented and reconstructured image of the new ultra-thin GDL.

In addition to SEM imaging, three-dimensional non-destructive scanning has been performed to obtain the details about ultra-thin GDL geometries using $\mu$-CT scanning. The details of the $\mu$-CT scanning method is explained in “Methodology and experimental procedure”. Using Avizo software, and Dragonfly software, segmentation and reconstruction of the image has been done. Figure 3 illustrates the results of the reconstructed images, where Fig. 3 illustrates the ultra-thin GDL by solid (light red) and pores (white) phases. Considering the mentioned governing equation, microstructural properties were calculated using the Avizo and Dragonfly softwares. Table 2 shows the results of the microstructural analysis obtained by the Xlab Simulation module by Avizo and the calculated porosity/thickness by the Dragonfly software. It should be noted that further microstructural properties such as water breakthrough and contact angle can be calculated in future studies using the presented microstructural properties in Table 2.

**Table 2:** Obtained microstructural properties using commercial software and reconstructed geometry by $\mu$-CT scan imaging.

Microstructural parameter	Ultra-thin GDL
Porosity	45.33%
Thickness	28.9 μm
Absolute permeability (μm^2^)	2490.4
Absolute permeability (day)	2523.4
Tortuosity	0.558
Apparent electrical conductivity (S/m)	1838.15
Apparent formation factor	3.767
Input concentration (mol/${\text{m}}^{3}$)	1711
Output concentration (mol/${\text{m}}^{3}$)	0
Apparent molecular diffusivity (${\text{m}}^{2}/\text{s}$)	0.0483
Apparent thermal conductivity $\left(\frac{\text{W}}{\text{m}.\text{K}}\right)$	10.148

Regarding the obtained microstructural properties of the ultra-thin GDL in Table 2, it should be noted that the calculated results are the outcome of the manufactured ultra-thin GDL using the proposed method (the novelty of this study) in “Methodology and experimental procedure”. The ultra-thin GDL is designed to resist compression forces of bipolar plates for up to 50 cells in a stack, however, in some settings higher cells are assembled in a stack. In this condition, the ultra-thin GDL can be used as a standalone MPL using metallic mesh GDLs as the supporter for the compression forces of the bipolar plates. Concerning the current density–voltage (I–V) characteristic curve, the overall performance of the PEMFC will be improved using the ultra-thin GDL following the fact that thinner GDLs lead to lower mass transport resistances, hence better I–V curve performances. As the typical GDLs in the market are around 90 to 200 μm, the ultra-thin GDL can provide improved microstructural properties and mass transport through GDL.

## Conclusion

After segmentation and reconstruction of the ultra-thin GDL sample, it was possible to obtain microstructural properties. The obtained results showed that the low pore size and the homogeneously distributed pores of the ultra-thin GDL enable good performance for this new product. It is believed that the current developed product could be efficient in theory, however, the production was always confronting cracking and deformation of powders. The novel methodology to produce the ultra-thin GDL prevented the formation of the cracks and the layer was formed without the formation of powders. The results of the microstructural properties also indicated that the novel developed GDL has better electrical conductivity, molecular diffusivity, and absolute permeability, but lower thermal conductivity.

Given the acceptable microstructural properties of the developed GDL and its low thickness, this product would enable more compact fuel cell/electrolyzer stacks, and lower consumption of materials to produce the GDL, which results in lower costs and lower environmental pollution. This novel GDL also reduces the overall weight of the stack that facilitates the use of stacks in mobility.

Although the proposed manufacturing method for the ultra-thin GDL in “Methodology and experimental procedure” followed by the microstructural analysis in “Results and discussion” were the main novelties of this study, future studies can be done to further confirm the suitability of this product and the improvement of GDLs in the future:

- Considering the pore diameter, the water breakthrough pressure can be evaluated with different GDL samples if there are disclosure agreement between the industrial partners and the research facilities. When the water breakthrough pressure is too high, liquid water would be accumulated at the catalyst layer which promotes flooding^23^.
- At the moment, the proposed ultra-thin GDL has been designed for the normal operating condition of a PEMFC. It is recommended to consider the improvement of this product for high current density conditions, which will impact the microstructural properties as well.
- This study was focused on the main purpose of developing novel ultra-thin GDLs without the consideration of GDL’s impact on the membrane dehydration. However, GDL is able to prevent membrane dehydration and improve the performance of PEMFC. In this regard, it is suggested to perform polarization curve measurements under low and high humidity conditions for future studies.
- Considering the achievement of this study to have low thicknesses of GDL, the performance of PEMFC will improve, however, it should be noted that further analyses should be done to characterize the oxygen supply to the catalyst layer under the separator rib regions.
- The current study used the EDXS results of the elemental mapping in addition to the SEM and $\mu$-CT observations for the size and the shape of the samples to calculate the microstructural properties using the Xlab module of the Avizo software. As a topic for future studies, it can be interesting to compare this method with other available options including 4-point probe to calculate the conductivity.

## Data Availability

The datasets used and/or analyzed during the current study available from the corresponding author on reasonable request.
